# Growth change in Polish women: Reduction of the secular trends?

**DOI:** 10.1371/journal.pone.0242074

**Published:** 2020-11-30

**Authors:** Monika Łopuszańska-Dawid, Alicja Szklarska

**Affiliations:** 1 Józef Pilsudski University of Physical Education in Warsaw, Faculty of Physical Education, Department of Human Biology, Warsaw, Poland; 2 Polish Academy of Sciences, Poland, Palace of Culture and Science, Warsaw, Poland; Universidad Autonoma de Madrid, SPAIN

## Abstract

The aim of the study was to analyse changes in the average height of adult Polish women born in 1931–2001 in the aspect of dynamically changing economic and socio-economic conditions of the living environment. An ethnically homogeneous group of 6,028 adult women from large Polish cities, born in 1931–2001, living between 1931 and 2020, were examined using the same research methods and research equipment. All women were divided into eight birth cohorts. The Kruskal-Wallis test and multiple regression analyses were used. Root Mean Square Standardized Effect (RMSSE), critical value of the test, and test power were calculated. The average height of women born during 70 years of the study increased by 9.63 cm, from 158.22 cm (SD = 5.57 cm) to 167.85 cm (SD = 6.91 cm) (H = 1084.84, p<0.001). The intensity of the intergenerational trend in subsequent cohorts of years of birth varied strongly between decades, averaging 1.34 cm/decade. The body height in women increased significantly up to the height of those born between 1970 and 1979 and then the trend weakened noticeably, although it remained positive. The observed secular trend confirms positive changes in the standard of living of Polish women between 1931 and 2020. Improving living conditions allow people to fully achieve their genetically determined growth potential.

## Introduction

Body height is a particular biological characteristic of humans, it is the basic anthropometric human phenotypic trait and it is of a cumulative character [1–3]. Furthermore, the broadly understood living environment of each individual, both in the prenatal period and for about the first two decades of life, modifies gene expression while allowing for a phenotypic response in the form of specific body height. Epigenetic factors are at the base of the mechanisms of gene expression regulation, and their importance has been proven by more and more research results in molecular biology [4,5].

An intensive increase in body height occurs during the first two decades of human life, especially during the critical periods in which the speed of growth is very intense and, consequently, affect adult height. These critical periods are the foetal stage, infancy (from birth to around two years), and adolescence (with growth spurt). In these periods, there is an increase susceptibility to nutritional deficiencies, illnesses, physical workload, and psychosocial stress that, finally affecting adult height and other anthropometrics and physiological variables. Secular trends and population differences in adult height and other anthropometrics and physiological variables are established by the material and emotional living conditions during infancy and, after the years of childhood (or second infancy) and the juvenile period, in a lower degree, during adolescence [6,7]. Only a slight growth is observed in the third decade of life, with stabilization of body height, and then there may be a slight decrease in this characteristic. Longitudinal studies have shown that both secular trends, as well as differences in adult height and other biological indicators in human populations, are established very early, at about two years of age [6], although there is also evidence of the influence of later childhood and adolescence on final adult height [7].

Over the last centuries, the mean height of adults has changed fundamentally, although unevenly in individual countries and without signs of clear convergence between them [8–10]. With complex interactions, evolutionary, genetic, and to a high degree, environmental factors, the longitudinal study of secular changes in body height mirrors the evolutionary processes and social and environmental conditions that have a direct effect on economic well-being and therefore nutrition and health, especially during the foetal stage, infancy and adolescence [10–13].

Long-term studies of adult body height trends have been conducted in Europe, the USA and Asia for at least 250 years. They have been mainly based on anthropometric data of men, most often conscripts, military personnel, convicted people, and previously even slaves [11,14,15]. In contrast to extensive research on secular trends in anthropometric characteristics of the male body, including Polish men [16–21], there is still a lack of comprehensive analyses of secular trends in adult female body height, including data on the Polish population. These studies are usually fragmented, based on a heterogeneous research methodology, and usually involve small research groups and short periods of time. Particularly little research has been done on the trends in women's body height changes in non-English-speaking and non-European parts of the world. Slightly more studies concerned the secular trends of biological characteristics in European women and those from the southern hemisphere [22–24].

Therefore, the present study aims to analyse changes in the average height of adult Polish women born after 1930 and attempted to explain the intensity and direction of the observed changes in this anthropometric characteristic.

## Material and methods

### Material

The material for the analysis consisted of data on 6,028 adult Polish women born within 70 years, in the years 1931–2001, and living in the period of 1931–2020. All women were Polish, and lived in large Polish cities (with over 500 thousand inhabitants). The data were collected by one research team over a period of nearly 50 years, from 1971 to 2019, within the framework of several research projects financed from various non-statutory grant funds (KBN—Committee for Scientific Research, MNiSW- Ministry of Science and Higher Education grants). The study was approved by the Council of the KBN/MNiSW in accordance with their recommendations concerning research with humans. The grant applications to the KBN and the MNiSW were reviewed and approved by a board of experts in many fields (including ethical standards). Before the study began the board took into account the observance of ethical standards in this human research. Only projects that received the highest criteria for substantive and ethical evaluation received funding. We did not try to obtain additional permits for our research, the grant application procedure did not require it. All procedures performed in studies involving human participants were in accordance with the ethical standards of the institutional and/or national research committee and with the 1964 Helsinki declaration and its later amendments or comparable ethical standards. All persons were orally informed about the aims of the projects and all testing procedures and all gave their informed consent prior to their inclusion in the study. At any time, the subjects could withdraw without giving any reason.

For the sake of clarity of the analysis, women were divided by year of birth into 8 birth groups (cohorts), namely: 1: 1931–1939, 2: 1940–1949, 3: 1950–1959, etc. All cohorts included births within 10 years (decade), except the first (9 years) and the last (2 years). The numbers of women in subsequent cohorts are given in Table 1. Women born between 1931 and 1979 (cohorts 1–5) were examined when they were 40–50 years old, whereas women born between 1980 and 2001 (6–8) were respectively younger at the time of the examination as they did not yet have a chance to reach the fifth decade of life. However, all of the subjects were examined at the age of over 18 years and therefore their process of fundamental growth had been completed. Years of examination, years of the adolescent growth spurt and the age of the women during subsequent examinations are presented in Table 1. The adolescent growth spurt refers to the rapid increase in height that begins with puberty, lasts throughout adolescence, and stops with the cessation of linear growth. The column *adolescent growth spurt* gives the range in the years when most subjects could expect a pubertal spike in body height (about 10–15 years of age).

**Table 1:** The number of Polish women in subsequent years of birth (N), women age (years), years of examination and years of adolescent growth spurt.

Group number	Years of birth	N	Age (years)	Years of examination	Adolescent growth spurt
			minimum-maximum		
1	1931–1939	**909**	40–50	1971–1989	1941–1954
2	1940–1949	**2401**	40–50	1980–2000	1950–1964
3	1950–1959	**1286**	40–50	1990–2010	1960–1974
4	1960–1969	**530**	40–50	2000–2019	1970–1984
5	1970–1979	**207**	40–50	2010–2019	1980–1994
6	1980–1989	**250**	30–40^	2010–2019	1990–2004
7	1990–1999	**399**	20–30^	2010–2019	2000–2014
8	2000–2001	**46**	18–19^	2018–2019	2010–2016
Total		**6028**			

^ these women have not yet reached the age of 40–50.

In all examinations, body height was measured by the team of expert anthropologists and anthropometrists, according to the same methodology and using the Martin technique [25,26]. Body height was measured as the distance of the vertex point from the basis (*B-v*: *Basis-vertex*) while maintaining the Frankfurt plane. Measurements were always made using the same certified testing equipment: Martin anthropometer (brand product: GMP Gneupel Präzisions-Mechanik, Swiss product) with an accuracy of 1mm. The measurements were taken in the morning. The participants were standing upright, without shoes and in their underwear. Although it is more time-consuming, body height measurements give much more reliable results than in the case of body height declared by study participants [27].

Although the collected research material is quite large, it does not constitute a strictly random sample of the female population of big cities, as it only contains information about people who volunteered and wanted to participate in the implemented grant projects. The average number of people participating in Polish examinations, including anthropological measurements, is about 25% [28]. The participants included in the analysis were not selected in any way in terms of anthropometric or other parameters. The study group was ethnically homogeneous, without linguistic or cultural minorities. The women participating in the study did not suffer from severe chronic diseases at the time of the study or in the past. No pathologies were also found in the women in the physical examination. The participants did not have any growth disorders and did not take drugs in childhood that could affect their adult body height.

Analysis of the changes in the average height of different groups of people over time reveals that this feature changes in different age groups in populations due to both secular and involutional changes. Data on the onset of body height changes are inconsistent and often contradictory. According to the results presented by Sikora [29], the mean body height is the highest around 20–25 years of age, it slightly reduces in older age groups, and it clearly decreases over 65–70 years of age, reaching a 4% difference between the youngest and the oldest people. Furthermore, research on selected somatic characteristics of Polish women aged 21–60 indicated the beginning of body height decrease at the age of over 30–35 years [30]. Slightly different results were obtained by Klaus [31], who showed that in women the intense loss of body height starts at around 60 years. Also the data on mean body height loss in ontogenesis are contradictory. The mean reduction in body height of women between 30 and 70 years of age ranges from 5 cm [32] to even 7.2 cm [33]. Therefore, it is important to analyse the effects of a secular trend in the relevant age cohorts so that the differences observed are not obscured by ageing processes [34,35]. Comparison of the above facts to the results presented here shows that, firstly, the analysis of changes in body height of women who are always in the same age group (in the 4th decade of their lives) in this paper, encourages drawing appropriate conclusions about secular trends. Secondly, research on women who may have already undergone some evolutionary changes and a reduction in body height further strengthens the findings of the study. It can be assumed that the maximum body height of these women was slightly higher.

### Statistical analysis

Changes in average height between years of birth (by cohorts) were computed and given in % of body height/decade and cm/decade (Table 2). Statistical significance of differences in body height between cohorts was estimated using the Kruskal-Wallis test. Root Mean Square Standardized Effect (RMSSE), a critical value of the test, and test power were calculated. Multiple comparisons of mean ranks for all samples were used to determine the significant differences in female height between decades of years of births. Multiple regression analyses were employed to assess the net effect of each year of birth on body height. Body height was a dependent variable, whereas the years of birth (as a continuous variable) were an independent variable. The values of determination coefficients R^2^ and adjusted R^2^ were given. They provide information about the percentage of the variance of the dependent variable explained by the determining variable. The level of significance was set at α = 0.05. The STATISTICA 12.0 and 13.5 packages were used for analyses [36,37].

**Table 2:** Means, standard deviation (SD), standard error of the mean, and gains in body height in eight years of birth’s groups of adult Polish women and results of Kruskal-Wallis’ test (H, p).

Group number	Years of birth	mean	SD	standard error of the mean	Gain % of height/decade	Gain cm/decade	Cumulative gain (cm)
1	1931–1939	158.22	5.57	0.1954	-	-	-
2	1940–1949	159.70	5.70	0.1221	0.93	1.48	-
3	1950–1959	160.66	5.88	0.1709	0.60	0.96	2.44
4	1960–1969	164.19	6.10	0.2596	2.15	3.53	5.97
5	1970–1979	166.57	6.76	0.4285	1.43	2.38	8.35
6	1980–1989	167.65	6.39	0.3810	0.64	1.08	9.43
7	1990–1999	167.73	6.53	0.2968	0.05	0.08	9.51
8	2000–2001	167.85	6.91	0.8686	0.07	0.12	9.63
**Mean gain**	**1931–2001**				**0.84**	**1.34**	**9.63**
**H, p**		**1084.84, p <0.001**					
RMSSE		3.9949					
Critical value of the test		2.0329					
Test power		1.0000 with α = 0.05					

## Results

Table 2 shows the means, and standard deviations of body height in subsequent cohorts, standard errors of the mean, and gains in body height in eight cohorts of years of birth of adult Polish women. The significance of changes in average height over time in the form of H test, statistical significance *p*, RMSEE, a critical value of the test and test power are also presented.

**Fig 1:**
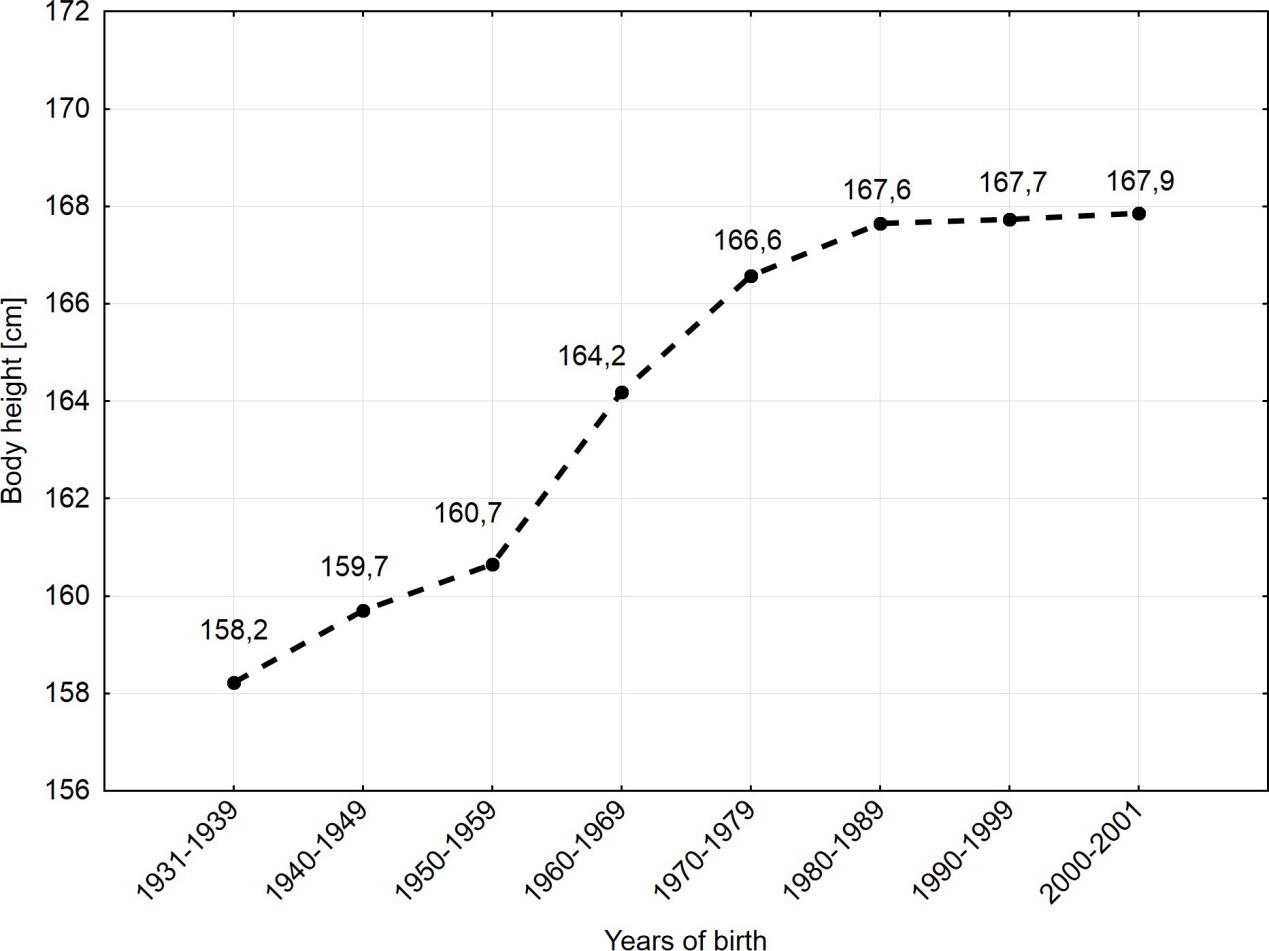
Secular trend in body height in women born in years 1931–2001.

The average height of the metropolitan women born between 1931 and 1939 was 158.22 cm (SD = 5.57 cm), and then gradually increased in subsequent cohorts to reach 167.85 cm (SD = 6.91 cm) in 2000–2001 (Table 2) (Fig 1). The observed positive intergenerational trend in women's body height turned out to be statistically significant (H = 1084.84, p<0.001), similar to the power of the applied test (η^2^ = 1.0000). The average height increased by 9.63 cm over 70 years, which is an increase of 6.09% compared to the average female height from the first birth cohort.

The intensity of the observed intergenerational trend varied over the following decades. The initially intense positive trend between the 1st and 2nd cohort (1.48 cm/decade) weakened slightly in the 3rd cohort (0.96 cm/decade). Then, in cohorts 4 and 5, a significant intensification of average female height gains was observed. Among women born between 1960 and 1969 and between 1970 and 1979, the gains were 3.53 and 2.38 cm/decade, respectively. Then, the trend towards greater body height clearly declined to around 1.00 cm in the 6th cohort, and it continued to slow down.

Multiple comparisons of mean ranks for all samples (Table 3) show that all differences in average height between cohorts 1-2-3-4-5 are statistically significant, while all differences in average height between cohorts 5-6-7-8 are no longer statistically significant. Therefore, the average height of adult women born between 1931 and 1939 increased significantly from decade to decade to the body height reached by women born between 1970 and 1979. The trend then clearly weakened and stabilized, although it remained positive.

**Table 3:** Multiple comparisons of mean ranks for all samples (p-values for multiple, two-sided, comparisons).

Group number		1	2	3	4	5	6	7	8
	Years of birth	1931–1939	1940–1949	1950–1959	1960–1969	1970–1979	1980–1989	1990–1999	2000–2001
1	1931–1939	-	**<0.001**	**<0.001**	**<0.001**	**<0.001**	**<0.001**	**<0.001**	**<0.001**
2	1940–1949		**-**	**<0.001**	**<0.001**	**<0.001**	**<0.001**	**<0.001**	**<0.001**
3	1950–1959			**<0.001**	**<0.001**	**<0.001**	**<0.001**	**<0.001**	**<0.001**
4	1960–1969				**-**	**<0.05**	**<0.001**	**<0.001**	0.0536
5	1970–1979					-	1.0000	1.0000	1.0000
6	1980–1989						-	1.0000	1.0000
7	1990–1999							-	1.0000
8	2000–2001								-

Table 4 presents the results of multiple regression analysis carried out for stature. Year of birth is an important determinant of changes in the stature of the women studied (<0.001). On average, for all years of birth, with each subsequent cohorts of the year of birth (period 1931–2001), adult women were 0.18 cm taller. The determining variable (year of survey) exhausts more than 20% of the body height variance.

**Table 4:** Results of multiple regression analyses of body height in Polish women.

	β	β standard error	B	B standard error	t	p
Year of birth	0.4522	0.0117	**0.1806**	0.0047	38.5950	**<0.001**
	R^2 = 0.2045; corrected R^2 = 0.2044; F = 1489.6, **p<0.001**					

## Discussion

The results indicate that the average height of adult Polish women born after 1930 has been constantly increasing. Mean stature has increased by 9.63 cm, which is significantly higher by ca. 1.5 standard deviations for that trait in the general population. This positive trend was observed throughout the 70-year-long study period, but its intensity was different in different decades of the period, with an average increase of 1.34 cm/decade, or 0.134 cm per year.

### International background

The increase in the average height of Polish women is similar to that observed in the last century for most industrialized countries [38] and cannot be explained only by the modification of the genetic structure of the population. Currently, the height differences between the shortest and tallest female populations in the world are about 20 cm and the differences remain almost unchanged. In men, the corresponding differences are about 19 cm and are constantly increasing [39]. The population of the United States, which had been the world’s tallest for nearly 200 years, was outgrown by the inhabitants of north-western countries such as Norway, Sweden and the Netherlands. In 2000, the average height of young adult women in the USA was 163 cm, which was about 7 cm (about 4%) less than in the Dutch population [40]. The intensity and speed of intergenerational changes in adult body height are now gradually decreasing in northern Europe and Italy [41–44], while the trend continues in some southern European countries, Spain and Portugal [41,45].

An international study conducted by the NCD Risk Factor Collaboration on 1,472 world populations [45], covering 18.6 million people born between 1896 and 1996, showed the highest mean height gain (20.2 cm; 95% CI:17.5–22.7) among South Korean women. In 1896, the shortest women lived in El Salvador, Peru, Bangladesh, South Korea and Japan. In 1996, the shortest women in the world remained in Guatemala (mean 149.4; 95%CI: 148.0–150.8), followed by Bangladesh, India and Nepal (mean 151.2 cm). The average height of women from Latin America and the Caribbean was 153.0–155.0 cm (14 studies from eight countries: Bolivia, Brazil, Colombia, Dominican Republic, Guatemala, Haiti, Nicaragua and Peru) [45,46], while for Central Asia, this was 156.9 cm (Armenia, Kazakhstan, Kyrgyzstan, Turkey and Uzbekistan). In 2006, the average female height of Turks increased to 158.9 cm (SD 6,4), which was 6.6 cm higher than the 152.3 cm recorded in 1937 [47]. According to the results of the NCD-RisC [45] research group, the average height of African women at the beginning of the 21st century was 157.8 cm (29 studies from 27 countries).

The tallest women in the world, about 20 cm taller on average than the shortest ones, live in Europe, mainly in its northern part: the Netherlands, Latvia, Estonia, the Czech Republic and Sweden, with an average height of over 168 cm. Currently, the average female height in Austria, Belgium, Germany, Finland, Ireland or the Czech Republic is over 165 cm, while the body height of Italian, Greek, Spanish or Portuguese women in southern Europe is around 165 cm [46,48]. A study by Cavelaars research team [49] on changes in body height in 10 European countries, conducted between 1987 and 1994 among women aged 20–74, showed similar trends. According to these authors, the tallest women live in the Netherlands, Norway, Sweden and Denmark, whereas the shortest—in France, Italy and Spain (from 160 to 167 cm).

### Secular trends and rate of height changes in Polish women

According to the NCD Risk Factor Collaboration data [45], Polish women, with an average height of about 166 cm, rank 33 out of 200 analysed countries worldwide. The results of this study indicated a slightly higher average height. Currently, the average height of adult Polish women is about 167.8 cm. Analyses of body height of young, 18-year-old women from various regions of Poland, carried out in 1966, 1978, 1988 and 2012 as part of subsequent Anthropological Photographs of Poland (although on much smaller research samples) also indicate an intensive secular trend in body height of young Polish women. Average height values in subsequent years were (respectively: year, mean, SD): 1966: 159.16, 5.44; 1978: 161.88, 5.73; 1988: 163.04, 5.73; 2012: 165.54, 6.22 [50]. Therefore, in the period of 1966–2012, the average height of the women studied increased by 6.38 cm, i.e. by 0.14 cm per year and 1.4 cm per decade. Also the results of this study, concerning over 6,000 adult Polish women, showed a significant intergenerational trend in body height. Average female height of Poles increased from 158.22 cm (1st cohort) to 167.85 cm in the last cohort, i.e. by 9.63 cm with an average intensity of 1.34 cm/decade. Other studies conducted on women born between 1933 and 1957 [51], among randomly selected from the general population of Krakow (Poland), Novosibirsk (Russia) and six cities of the Czech Republic, showed that the average height of women was 159.4 cm (SD: 5.9 cm) in Poland, 158.2 cm (SD: 6.0 cm) in Russia and 161.9 cm (SD: 6.1 cm) in the Czech Republic [51]. Furthermore, the authors showed a change in the average height of women born in successive 5-year-old cohorts. For the population of Polish women, the changes were as follows (respectively: cohort, mean in cm, SD in cm): 1933–37: 157.1, 5.5; 1938–42: 158.2, 5.6; 1943–47: 159.3, 5.6; 1948–52: 160.2, 6.0; 1953–57: 161.4, 5.6. In Russia, changes in the same period were from 155.6, 5.5 to 160.8, 5.8, respectively, whereas in the Czech Republic, from 159.2, 5.7 to 164.3, 5.8 respectively. Therefore, the mean height increase among Polish women was the lowest (0.4 cm per decade), while in Russia, it was the highest (1.1 cm per decade) [51]. Subsequent studies of Polish women showed mean and standard deviations in body height of girls from cities of Upper Silesia region of Poland in 1964, 1974, 1986 [52]. The authors demonstrated the presence of a positive trend in body height of the women studied (1964: 160.0, 5.5; 1974: 160.4, 5.6; 1986: 163.0, 6.3). In this case, the mean change per decade was 1.36 cm. Therefore, the secular changes in the body height of the Upper Silesian population, which have been observed for twenty years, and which also serve as an indicator of the economic situation, indicate its general improvement.

Previous trend studies of the Polish population have estimated the rate of secular changes to be about 0.1 cm per year for older age groups (1903–1923) and about 0.2 cm per year for younger age groups [53,54]. The most recent studies of the Polish female population indicate a comparable (although quite variable) rate of increase in body height in subsequent periods, from about 1.36 cm/decade in 1964–1986 [52], 1.4 cm/decade in 1966–2012 [50], to 2.15 cm/decade found in the present study for women born in the 1960s.

The rate of the secular trend for body height observed among Polish women is comparable to that observed in other female populations at that time. Data on Russians from 1705–1945 indicate an average increase in average female height of about 2 cm/decade [55]. In the same period, Brainerd (unpublished data [51]) estimated the average height gain of Russian women at 1.7 cm/decade. Furthermore, the study of women from 1946–1972 living in St. Petersburg, showed an increase in the average height of women by 2.4 cm/decade [56]. Cavelaars et al. [49] showed a slightly slower trend. According to these authors, average height increased linearly with the year of birth at a rate of 0.4 cm/5 years.

In less economically developed countries, the observed rate of trends in average female height is significantly lower and oscillates around 0.5 cm per decade (Armenia, Moldova) following the only 0.22 cm per decade in Indian women [57–59]. The trend rate is even lower among women from the least developed countries [60]. An international study conducted in 54 underdeveloped countries, covering a representative sample of 364,538 women aged 25–49 from 1994–2008, showed an average annual height gain of 0.0138 cm (95%CI: 0.0107, 0.0169) [61,62].

### Polish background

Over the last century, social, economic and environmental conditions in Poland were extremely unstable and highly varied. It is known that economic processes have a strong modifying effect on biological processes [63], shaping and modifying the biological condition, health status and mortality rate of the populations concerned, which was also found for the Polish population [64–68]. In the following years from the period between the 1930s to the 1920s, Polish population experienced dramatic socioeconomic, environmental and nutritional changes. Poland has seen several successive economic crises and periods of dynamic change. These included the economic crisis of the Great Depression (1930s) and the Second World War (WWII, 1939–1945), the period of communism (1945–1989), dramatic changes in the transformation of the political system (1989-1997/2004) towards capitalism until Poland joined the European Union (2004), and the opening of EU labour markets. All this undoubtedly had an effect on the physical features of this population [69].

Since secular trends towards greater body height are mainly caused by the progress of civilisation and the improvement of living standards, the differences in body height may indicate that the living conditions of women from individual cohorts vary, mainly during the growth process. the critical periods on human life cycle in which the speed of growth is very intense, especially in infancy and adolescence.

The period of intensive growth in infancy and adolescence of the women of the first two cohorts fell during a very difficult period of strong crisis of the Great Depression and World War II, so low body height values in women seem obvious. Some researchers point to the ruinous consequences of the Great Depression for both the economy and the living standards of Polish society [19]. They state that the twenty years period between two world wars allowed only to rebuild the economic potential of Polish lands to the level reached previously in 1913 r. Adverse living conditions in Poland during the Great Depression is corroborated by the Committee for Nutrition of the League of Nations Report in 1936, which states large-scale malnutrition. In the Great Depression, the consumption of basic food products per capita decreased significantly, e.g. between 1929 and 1933, the consumption of sugar fell from 11.9 kg to 8.6 kg, meat from 18.4 kg to 18.3 kg (through 17.6 kg in 1930), rice from 1.2 kg to about 1.0 kg, yeast from 0.3 kg to 0.2 kg. Compared to other countries, Poles consumed much more rye bread, flour, potatoes, peas and beans, while definitely less the more valuable products like meat, fish, groats, fats, milk, butter, cheese, eggs, vegetables and fruit. The basic costs of living for families, such as rent, fuel, lighting, transport, postal services, increased, while the prices paid to producers for crops fell by as much as 70%. The retail prices of basic necessities increased by up to 100% in relation to the period before the Great Depression (e.g. wheat flour from PLN 58/kg in 1924 to PLN 86 in 1930, pork from PLN 207/kg to PLN 314, milk from PLN 37/l to PLN 45). In 1929–34, unemployment in Poland increased by 314% [70].

The impact of political and economic conditions in Europe during WWII on the biological status of human populations has recently more studies [71,72]. The standard of living during World War II deteriorated significantly. The Polish population was exposed to long-term nutritional stresses, increased disease incidence, poorer hygienic conditions and multi-faceted long-term stress related to the reality of war. Food coupons for many basic food items were introduced by the occupant. Research on the Polish population indicates that selected biological characteristics, commonly used as a “barometer” of human well-being, achieve significantly worse values during WWII than before them, e.g. students born during the WWII are shorter than students born before WWII, or age at menarche increased among women born during WWII compared to those born before [71,72].

Post-war period and the 1950s initiated long-term economic growth, the standard of living, especially of families and children, has improved and brought about an increase in the average height of Polish women by about 1.5 cm/decade. For the first time ever, the plans to improve the economic system were based on taking into account social needs. The number of private businesses was also growing, and commercial and catering facilities and craftsmen's workshops were established. Problems in the food market and low wages were compensated by the authorities by financing health care, education, culture, subsidies for the rent, heating, energy, public transport, train commuting and holiday trips [68,69].

The strongest trend towards greater growth was found (4th and 5th cohort) in women born in the 1960s and 1970s. The women born between 1960 and 1969 and between 1970 and 1979 outgrew their peers from previous decades by 3.53 and 2.38 cm, respectively. The causes for this situation are to be found in the living conditions of the 1960s and 1970s. The most important periods on this women life, affect their adult height, occurred in the years when national income increased by almost 60%, industrial production rose by 64%, agricultural production by 19%, and real wages by 41%. Products which were previously considered to be luxurious appeared on the market at affordable prices, e.g. chocolate goods, citrus, high-quality sausages, etc. All this is due to state borrowing, the use of foreign currency reserves, and the increase in demand for coal. The results obtained for the female population are confirmed by those obtained for the Polish male population for which intensive positive secular trends were found for the decade, 2.4 and 2.0 cm, respectively [14].

The social and economic changes in Poland in the 1980s contributed to the rapid weakening of the intensity of the trend towards greater body height. The secular trend in the body height of women born between 1980 and 1989 slowed down by more than 50%, and reached a level lower than after World War II. For this cohort, the first two years of life coincided with one of the worst political and economic crises in the history of Poland initiated by the introduction of martial law in 1981. The drop in real household income reduced spending on quantitatively and qualitatively improving nutrition, medical care, and leisure. There were dietary restrictions in Poland and the multi-assortment food coupons were in force. There was a drop in consumption of lean meat and fresh fruits and vegetables, which were largely replaced by starchy foods [14,73]. The economic collapse also resulted in diametric changes in lifestyle, such as an increase in alcohol and tobacco consumption [14,74]. Very similar inhibition of the trend towards greater body height was found in the population of Polish conscripts, whose growth in this decade was even lower than in women and amounted to 0.8 cm/decade [14]. The researchers explained that the slower increase in the rate of growth may be interpreted as a combination of prepubertal stunting combined with late catch-up growth [14,75].

In the following years, despite the still positive trend of changes, there was only a slight acceleration of body height. Although the adolescence of women born after 1990 occurred in favourable years (e.g. Poland's accession to the EU, the opening of EU labour markets), the secular trend of body height weakened noticeably, although it remained positive. The main reason was that their the main critical period for adult height (about first two years of life) coincided with the initial "shock treatment" phase of the political and economic transformation (1989–1993). In the same period Kołodziej et al. [14] and Lipowicz et al. [17] recorded a very weak positive trend in body height (gain +1.0 cm/decade), while trend of increasing BMI was halted. During this period, there was a large drop in Gross National Product (GNP), a large increase in unemployment, and hyperinflation that reached as high as 586% in 1990. Real household income decreased, and households curtailed spending on food, with both the quantity and quality of food consumed decreasing. Families also spent less on recreation and medical care. There was a decrease in the consumption of lean meat and fresh fruits and vegetables, and an increase in the consumption of starch [17]. A number of other studies on the biological condition of the Polish population during the period of dynamic political changes and the accompanying economic and social changes have emphasized an increased hazard behaviour and noticeable deterioration in health and an increase in the mortality rate, regardless of the development period of individuals [14,65–67,71,72,76–78].

In general, the average intensity of the body height trend of Polish women compared to neighbouring countries can be considered high. This is confirmed by Ivanova's et al. results [79], who found the acceleration of the pace of changes in the somatic body structure of the inhabitants of countries that have undergone the transformation with relatively greater success, such as the Baltic States, Poland, the Czech Republic or Slovenia. They found a slightly lower rate of secular trends in countries where systemic changes have not brought the expected level (e.g. Romania, Bulgaria, some countries of the former Yugoslavia). For example, the slower pace of change in Bulgaria is explained by the sharper crisis and a much slower rate of economic growth compared to Hungary or Poland [79].

It is known that the final body height of an individual is influenced by the genotype and certain environmental stimuli such as the amount and quality of nutrition in relation to the body's energy expenditure and the degree of burden of certain diseases in infancy and adolescence [7,80]. Recent studies on the determinants of body height changes in the population of European origin indicated that the most important factor explaining the current differences in body height is the level of nutrition, in particular the ratio between the intake of high-quality proteins from dairy products, pork and fish and the consumption of lower quality proteins from wheat [38]. In the similar period in Poland, the consumption of meat and offal increased almost five times, from 15.8 kg per capita in the 1940s, to 74.3kg in 2010 [81,82], which could be related to an increase in average height. However, both the quality of nutrition and the burden of disease from birth to around two years, and in adolescence are environmental factors mainly related to the social and economic situation of individuals and their families, which are an important part of the standard of living. Therefore, intergenerational changes in body height can be an interesting source of information about changes in living standards over time. The results of studies on the intensity of the secular trend in body height of nearly 150,000 nineteen-year-old conscripts indicated temporary slowdowns and accelerations reflecting periods of crisis and acceleration of the country's economic development. Thus, the slowdown in the intensity of changes in the period between 1986 and 2001 is explained by the pauperisation of the lives of large social groups [11,83]. The analysis of changes in the secular trend of adult body height of Polish women shows similar, although not identical, fluctuations in trend intensity as in the case of conscripts. Slight time shifts in periods of trend slowing down or its intensification should be explained by the different timing of critical moments of ontogenetic development of 19-year-olds and 40-50-year-old women. The significant study limitation may be the fact that the surveyed women come from large Polish cities and do not represent small city and rural area. As indicated by the research of Polish conscripts, in Poland there are a significant social gradients of body height, which additionally remain exceptionally stable and unchanged, despite the improvement in the standards of living of the entire society [18,63]. It can therefore be expected that the body height of Polish women from areas with a lower degree of urbanization is significantly lower, but the secular trend is also present.

### Reversing the trends?

There is currently an international scientific discussion on the possible reduction of the secular trends in biological characteristics [84]. This results from the reduction, in some countries, not only in northern Europe, the trends towards greater body height, and sometimes also reversing these trends. In sparsely populated countries (e.g. Azerbaijan) temporary economic difficulties may be the cause. In Lithuania and other countries of the former Eastern Bloc, the economic recession of the early 1990s also led to the reduction in mean body height of 18-year-olds, but only among boys [85]. In 2001, the mean body height of 18-year-olds was 181.3 cm in boys and 167.5 cm in girls, and in 2008, this was 179.7 cm and 167.9 cm, respectively [86]. Because Lithuania's economic situation has improved significantly over the last 15 years, the modern generation of teenagers could exceed the level from 2001.

There is no uniform scientific explanation for the biological mechanism of the positive trend in female body height. On the one hand, it is claimed that it is caused by the increasing length of the lower limbs and sitting height (*Bs-v*: *Basis sedilis-vertex*), and on the other hand, there is evidence that the increasing length of the lower limbs is more responsible for the presence of a trend [24,87–90]. However, there are also indications that the characteristic that is most plastic and most sensitive to external conditions is total body height and, to a much lesser extent, the length of the trunk or lower limbs [91].

## Conclusions

Environmental regulators play a fundamental role in the expression of genes and the determination of traits and phenotypes. Fluctuations in the intensity of the secular trend in body height in Polish women from big cities are quite similar to the slightly less intense (also slowing down, but still positive) secular trend in the population of young male Poles [14]. However, the lack of clear convergence of the economic situation with the curve of the intensity of trends in men is explained by the authors by the hypothesis related to the protective role of the family and complex social support systems. Perhaps at the root of the biological difference in the intensity of the secular trends of both sexes are the person’s sex and sex hormones and/or environmental regulators in the form of varied lifestyles and strategies for dealing with chronic environmental stress. On the one hand, the ecosensitivity of male body is greater, but on the other hand, the substantially greater willingness of women to participate in preventive care, follow the recommendations for health practices and less involvement in high-risk behaviours may be associated not only with a reduced risk of morbidity or mortality but also with phenotypic expression of certain somatic characteristics different than that of men. Compared to men, women seem to have less ecosensitivity of body height, but their healthier lifestyles allow them to achieve a higher intensity of the trend with the same quality of living conditions. Sex and sex differences play a significant role in the development of body height and intensity of secular trends, but they have not yet been fully explored in detail and more research is needed in this area.

## Supporting information

S1 File (XLSX)
